# Varieties of male-sexual-identity development in clinical practice: a neuropsychoanalytic model

**DOI:** 10.3389/fpsyg.2014.01512

**Published:** 2014-12-22

**Authors:** Frans Stortelder

**Affiliations:** ^1^Department of Child and Adolescent PsychiatryGGZinGeest, Amsterdam, Netherlands; ^2^Supervising Analyst, Dutch Psychoanalytic Training InstituteAmsterdam, Netherlands

**Keywords:** neuropsychoanalysis, male homosexuality, masculine identity, procreative identity, alienated self, internalized homophobia, psychoanalytic relationship

## Abstract

Variations of sexual identity development are present in all cultures, as well as in many animal species. Freud – founding father of psychoanalysis – believed that all men have an inherited, bisexual disposition, and that many varieties of love and desire are experienced as alternative pathways to intimacy. In the neuropsychoanalytic model, psychic development starts with the constitutional self. The constitutional self is comprised of the neurobiological factors which contribute to sexual identity development. These neurobiological factors are focused on biphasic sexual organization in the prenatal phase, based on variations in genes, sex hormones, and brain circuits. This psychosocial construction of sexual identity is determined through contingent mirroring by the parents and peers of the constitutional self. The development of the self—or personal identity—is linked with the development of sexual identity, gender-role identity, and procreative identity. Incongruent mirroring of the constitutional self causes alienation in the development of the self. Such alienation can be treated within the psychoanalytic relationship. This article presents a contemporary, neuropsychoanalytic, developmental theory of male-sexual identity relating to varieties in male-sexual-identity development, with implications for psychoanalytic treatment, and is illustrated with three vignettes from clinical practice.

## INTRODUCTION

Varieties of sexual identity development are present in all cultures and in many animal species. Sexual fantasy, behavior, and identity become differentiated during the development of sexual orientation. Freud himself – founding father of psychoanalysis – believed that all men have inherited-bisexual dispositions, and that many varieties of love and desire are experienced as alternative pathways to intimacy (Cohler and Galatzer-Levy, 2000). Sexual fantasy is a core feature of both homosexuality and heterosexuality (Friedman and Downey, 2008). It was in the early 20th century that the concept of homosexual identity developed, integrating homosexual fantasy with homosexual behavior and identity (Cohler and Galatzer-Levy, 2000). Only recently, however, did homosexuality become an acceptable, socio-cultural category of sexual identity within modern, Western culture.

How does sexual identity develop? Psychic development begins with the constitutional self. The constitutional self comprises the neurobiological factors contributing to sexual identity development. These neurobiological factors are focused on biphasic-sexual organization in the prenatal phase, based on variations in genes, sex hormones, and brain circuits. This psychosocial construction of sexual identity is determined through contingent mirroring by the parents and peers of the constitutional self. The development of the self—or personal identity—is linked with the development of sexual identity, gender-role identity, and procreative identity. Incongruent mirroring of the constitutional self causes alienation during self development. Such alienation can be treated within the psychotherapeutic relationship by the psychoanalyst.

This article presents a contemporary neuropsychoanalytic-developmental theory of male-sexual identity relating to varieties of male-sexual-identity development, while offering implications for psychoanalytic treatment, and is illustrated by three vignettes from clinical practice.

## THE NEUROPSYCHOANALYTIC MODEL

Neuropsychoanalysis links models for psychoanalytic and neurobiological development through interdisciplinary perspective. Neuropsychoanalysis proposes that brain development and psychic development are interrelated. Changes in brain structure and function go together according to the structure-function principle (Schore, 1997). In the 20th century, psychoanalysis developed as a clinical science with a changing neuropsychoanalytic perspective. Unifying mind-body concepts evolved, from Freud’s “drive-concept”—which bridged biological and psychic drives—to the “energy-concept” of Ego Psychology—which connected psychic-energy processes with neurophysiologic-energy processes in the brain. Contemporary psychoanalysis is neuropsychoanalytic in its use of a unifying “representation concept.” The representation concept connects mental representations with neural representations, e.g., visual or motor, in the brain (Stortelder and Ploegmakers-Burg, 2010).

The neuropsychoanalytic model adheres to the bio-psychosocial view that brain structure and function are programmed by interpersonal experiences, resulting in the development of the psyche. Psychic development, which starts with the constitutional self, comprises inborn personality characteristics including sexual identity, constitutional developmental capacities, as well as genetic-risk factors for the development of psychopathology (Gergely, 2007). The self must take an active role, as a mental agent interacting with its environment, to socio-culturally construct its own personal and sexual identity.

The neuropsychoanalytic model also adheres to the ontogenetic principle in the evolution of living systems. Early development creates the foundation for the functioning of an organism for the rest of its life. New organization recapitulates and develops out of previous stages. Likewise, according to the ontogenetic principle of psychoanalysis, the development and organization of basic, psychic functions occur in the first 5 years of life, while a re-organization takes place during adolescence. Neurobiological research confirms that the basic growth and maturation of the brain occurs during the first 5 years of life, while a substantial reorganization in brain development transpires during adolescence (Stortelder and Ploegmakers-Burg, 2010).

Our article, “adolescence and the reorganization of infant development: a neuropsychoanalytic model” (Ibid), presented a neuropsychoanalytic-developmental theory which distinguished between psychosexual, emotional, and cognitive developmental domains, along with the domains for the development of self-agency and self-concept. Here is a brief review of the neuropsychoanalytic model for the development of self-concept or identity—as specified by sexual identity—in which early neurobiological development must be considered.

## NEUROBIOLOGICAL MODEL OF SEXUAL IDENTITY DEVELOPMENT

The development of sexual identity starts with the constitutional self. In the current view of sexual identity development, inborn-neurobiological factors play an important role. The neurobiological model of sexual identity development consists of gene-expression, neurochemical and brain development.

### DEVELOPMENT OF GENE-EXPRESSION

From behavioral genetic studies, it is known that homosexuality runs in families. Studies of twins show that among pairs of male-monozygotic twins, 52% are both homosexual; among pairs of male-dizygotic twins, 22% are both homosexual (Bailey and Pillard, 1991). In a study of adult sons of gay fathers 9% showed exclusive homosexual orientation (Bailey et al., 1995). In a longitudinal study of 17-year-old adolescents—an age where sexual identity is not yet fully crystallized—who were reared in lesbian families, 0% of the girls and 5.4% of the boys identified as homosexual, while 18% of the girls rated themselves on the bisexual spectrum (Gartrell et al., 2011). In an average population, approximately 3% of females and 4% of males express exclusively homosexual desires. The percentage of homosexuality may increase through genetic transmission from parent to child. However, the children of homosexual parents grow up predominantly identifying as heterosexual (Tasker and Patterson, 2007).

Along with other personality traits, the heredity of human-sexual identity is thought to be polygenetic, determined by multiple genes. Separately these genes have a low chance for expression. Combined in one carrier, they may surpass a threshold leading to homosexuality. The search for homosexual genes resulted in the finding of the Xq28 gene, transmitted across the maternal side of the family (Hamer and Copeland, 1994).

### NEUROCHEMICAL DEVELOPMENT

Neurochemistry includes the study of neurohormones and neurotransmitters. Most research, concerning neurochemical influence on the development of sexual identity, focuses on the Prenatal Androgen Theory. The theory suggests that genetic variation leads to variation in the influence of prenatal androgens. Such variation determines sexual differences within the brain in terms of gender identity (maleness and femaleness), gender-role behavior (masculinity and femininity), and sexual identity (erotic fantasy and desire for intimate relationship).

The chromosomes XX cause the embryogenesis of female genitals through the Mullerian duct, and the chromosomes XY of male genitals through the Wolffian duct. The Y chromosome activates a gene, the testis determining factor, which begins developing the testes. The testes start releasing the male sex-hormone testosterone, masculinizing the Wolffian system, so that male genitals may develop. In a female fetus lacking the Y chromosome, the Mullerian Duct develops into female genitalia. The prenatal androgens determine the development of maleness. If there are no prenatal androgens, the fetus will be female.

Prenatal sexual organization has a biphasic course, beginning with the sexual differentiation of the genitals occurring during the first 2 months of life. Sexual organization of the brain occurs during the second half of pregnancy. Its activation becomes overt, in the postnatal phase throughout the first 5 years of life, as well as during puberty. The Prenatal Androgen Theory postulates that male homosexuality is due to variation in levels or function of prenatal androgens, during the prenatal-masculinizing-brain development in the second half of pregnancy. Variation in androgen levels or function may only be present prenatally. Among adults, there is no difference between the sex-hormone levels of homosexuals and heterosexuals (Bao and Swaab, 2010).

### BRAIN DEVELOPMENT

Within the brain, psychosexual function is primarily organized by the hypothalamus, which regulates other vital bodily functions such as hunger, body temperature, circadian cycle, and stress. Brain research, relating to genetic variation and variation in the function of prenatal sex-hormones, focuses on structural variations of the hypothalamus. These hypothalamic structures organize sexual motivation and orientation, especially the sexually dimorphic nucleus of the pre-optic area. Swaab and Hofman (1990) found that the interstitial nucleus of anterior hypothalamus 1 (INAH-1) in the hypothalamus of homosexual men is intermediate in size between the hypothalami of heterosexual men and heterosexual women. LeVay (1991) discovered that the size of INAH-3 in homosexual men is smaller than in heterosexual men, while larger than in heterosexual women.

Thus, the constitutional self of sexual identity development has a multifactorial determination. The constitutional factors attributed to sexual identity development are not determined by a sole gene, hormone, or brain localization, but dependent on multiple genetic, neurochemical, and neural networks. Further research on the complex biodiversity of human-sexual-identity development is needed.

## PSYCHOANALYTIC MODEL OF SEXUAL IDENTITY DEVELOPMENT

In the psychoanalytic model, the development of sexual identity is the result of the interaction between the constitutional self and psycho-social experiences. Our article “adolescence and the reorganization of infant development: a neuropsychoanalytic model” presented a psychoanalytic model of psychic development, discussing the psychic dimensions of psychosexual development, relating to emotional and cognitive development, and development of the self. The development of the self is primarily determined by the formation of the self-concept. The self-concept comprises the self as an object (“Me-self”), and must be distinguished from self-agency or the self as a subject (“I-self”). What follows is a review of the psychic development of the self-concept, or personal identity, specified by linked domains of sexual identity, gender identity, and procreative identity.

### THE DEVELOPMENT OF PERSONAL IDENTITY

The development of a conscious sense of self starts by age one-and-a-half when the toddler recognizes itself in a mirror. Representations of the self-concept are concrete during childhood; in adolescence they become more abstract and more dependent on romantic attractiveness, as well as status at school and work (Harter, 2012). The development of self-representations is determined mostly during early childhood in the mirroring interaction with parents. During latency and adolescence, it is also determined by the mutual affirmative interactive process with peers and school (Ibid).

Fonagy et al. (2002) describe a psychoanalytic model of the mirroring process through the mechanism of affect mirroring. Affect mirroring consists of four aspects: the focal attention to and recognition of affect; the interpretation and representation of affect; the modulation of affect; the expression of an emotional response or action. In Fonagy’s model, the infant finds its primary emotional state represented by the markedly mirrored affect representation of the caregiver, decoupled from the realistic, affect representation of the caregiver, as well as anchored within itself as a secondary-affect representation. The infant is looking for a high-but-imperfect, contingent representation of its own affect in the mirroring caregiver, using a contingency detection module.

The interpretation of the infant’s affect by the caregiver forms an essential part of the process of affect mirroring. For example, if an infant cries from hunger, the contingent-mirroring caregiver says: “you are crying (affect recognition). You are hungry (affect interpretation/representation). Just be quiet (affect modulation). You get a bottle (emotional response/action).” A congruent, secondary-affect representation develops in the infant, along with a contingent, self-to-other, causal-emotional scheme. In the mirroring process, the infant has an active role as a mental agent through contingency detection. This means that his crying stops when he gets the expected response. However, the non-contingent, mirroring caregiver says: “you are crying. You are tired. Just be quiet. You are going to be put in bed to sleep.” The infant gets an incongruent mirroring of its affect and a non-contingent response. The incorrect interpretation of its caregiver leads to the development of a non-authentic or alienated, secondary affect-representation with a distorted, other-to-self causal-emotional scheme (Gergely).

**FIGURE 1:**
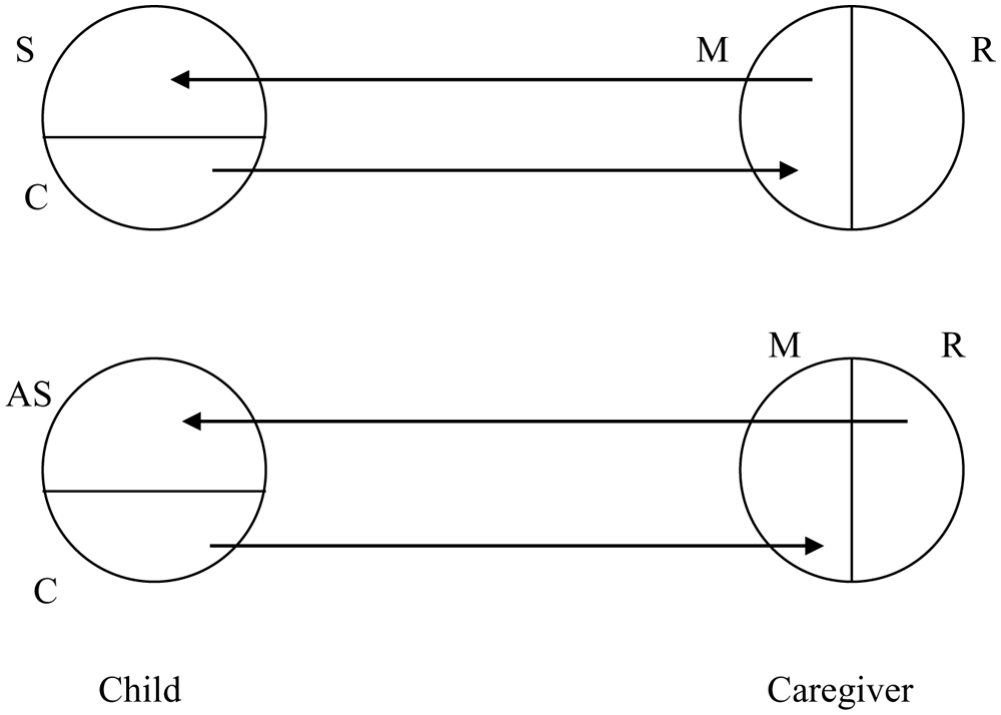
**The Affect Mirroring model: in the child, secondary self-representations (“S”) of the constitutional self (“C”) are formed through the contingent mirroring self (“M”) of the parent, decoupled from his realistic self (“R”).** In the case of incongruent mirroring by a realistic self-representation (“R”) of the parent, alienated secondary self-representations (“AS”) are formed in the child (Stortelder and Ploegmakers-Burg, 2010).

The following model (**Figure 1**). accounts for the mirroring of mental states leading to the development of mental representations, and of self-states leading to self-representations. It shows the development of an autobiographical self. This occurs through the parents’ congruent mirroring of the authentic self of the child. Incongruent mirroring of the authentic constitutional self leads to the development of an alienated self. In the neurobiology of the mirroring process, mirror neurons play a role in the implicit, simultaneous resonance within the self, reflecting the caregiver’s intentions (Gallese, 2009).

**FIGURE 2:**
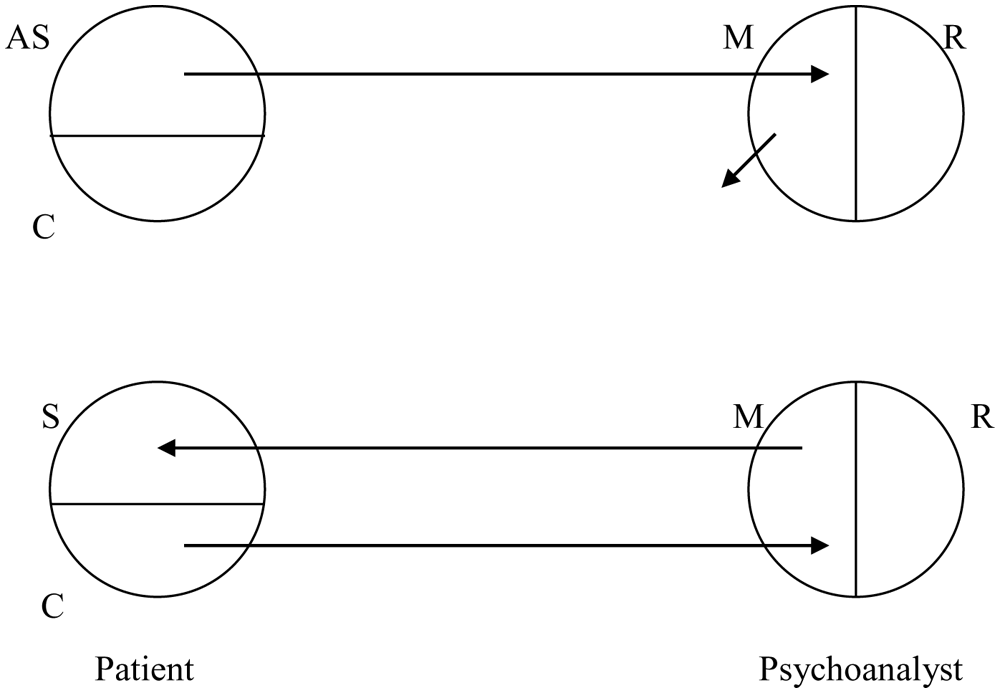
**The treatment of the Alienated Self: first, unconscious alienated self-representations (“AS”) of the patient are made conscious, in the transference-countertransference process, by the marked mirroring of the psychoanalyst (“M”), decoupled of the realistic self (“R”), and interpreted, so that they can be controlled and rejected by the patient.** New, contingent secondary self-representations, of the previously defended authentic self of the patient, are developed through the therapeutic mirroring of the psychoanalyst (Ibid).

Psychoanalytic treatment aims to change the non-authentic or alienated self. In the alienated self, there is an unconscious conflict between alienated self-representations and the defended, authentic self. During psychoanalytic treatment, these unconscious, alienated self-representations are brought into the transference by the patient, then marked and made conscious in the mirroring counter-transference by the psychoanalyst, and is decoupled from the psychoanalyst’s realistic self-representations (**Figure 2**). Such alienated self-representations are interpreted so they can be controlled and rejected. During this process, counter-transference consists of two aspects: representations originating from the transference of the patient, and realistic representations originating from the psychoanalyst. If the psychoanalyst mirrors a realistic self-representation, the mirroring is non-contingent and counter-transference becomes pathological.

In the phase of ‘working through,’ the psychoanalyst serves as a developmental object, stimulating the development of new, contingent, secondary self-representations of the previously defended, authentic self by the patient. The patient has an active role, through contingency detection, in the psychotherapeutic process. The patient reacts positively, feeling recognized and affirmed by the congruent mirroring of the psychoanalyst.

The development of an adult-personal identity is a core task during adolescence. Adult identity denotes a sense of consistency and continuity of the self, as well as a sense of personal norms and ideals. Erikson (1968) regards adolescence as an experimental period (“moratorium”), for discovering and building the various aspects of the authentic adult identity. Identity formation occurs, particularly in middle adolescence, by contact with like-minded peers, who validate and admire each other while sharing joint activities and ideas. During this mutual, mirroring process, mirror neurons play an essential role in the formation of individual identity—in its relation to social identity—through social identification (Gallese).

Music and clothing styles serve as identity markers and can be regarded, during treatment, as a window into an adolescent’s internal world. There are innumerable social groups, including skaters, rebels, punks, emos, and goths. Online role-playing fantasy games, such as *World of Warcraft* or *Still Life* can provide the adolescent with a virtual-transitional space to play with new identities. In identity formation, it is important for the adolescent to have a private space, to pose as a temporary outsider free from the rules and restrictions of the parental home. The adolescent needs to search for his own authentic identity based on his own experience, and by mirroring his peers. Adolescents should be able to fall back occasionally on the secure relationships of the parental home, while retaining the opportunity to experiment in the future, adult world. For homosexual adolescents, the LGTB community can offer such a private space (Stortelder and Ploegmakers-Burg, 2010).

### DEVELOPMENT OF SEXUAL IDENTITY

Friedman and Downey (2008) present an extensive historical overview of psychoanalytic theories of sexual identity. Indeed, they propose a contemporary psychoanalytic model of psychosexuality. In their design, psychosexual development starts in the prenatal stage under the influence of sex chromosomes. The development of the hypothalamic-pituitary-gonadal axis (HPG–axis) and the production of the sex-steroid hormones begin this process. These determine the embryogenesis of the reproductive system and the prenatal programming of the sexual brain, with the organization of psychosexual development during the first 5 years of life, as well as the activation of the HPG–axis at the onset of puberty.

After about 6 months, infants start to touch their genitals and experience erotic arousal (Roiphe and Galenson, 1972). Childhood masturbation is especially frequent between ages two through four. Starting around age four, erotic feelings and fantasies develop within a romantic, oedipal scenario. The inner narratives of these intrafamilial relationships become unconscious mental scripts for later, extra-familial relationships (Friedman and Downey, 2008).

Isay (1989) points out one way in which varieties in sexual identity development may be encountered in clinical practice. The pre-homosexual boy may develop unconscious romantic feelings and fantasies involving same-gender peers by kindergarten age, and may also have feelings directed toward his father. The pre-homosexual boy distances himself from his same-gender peers and father in order to avoid recognition of his romantic attachment. The boy’s self-perception of these silent, same-gender, erotic emotions may account for feelings of self-isolation and alienation. In return, the father may become detached and hostile toward his son. The father may unconsciously sense the son is different from other boys, as well as the son’s need for closeness and romantic attachment.

The father’s withdrawal is experienced as rejection by his son, along with a sense of masculine inadequacy. The father may present himself as a role model, and try to enhance heterosexual and masculine development in the son. Why? He believes that doing so will improve his son’s popularity with peers. Unconscious conflict develops between the alienated, self-representations and the defended, authentic self of the son, due to incongruent mirroring by the father. Such unconscious conflict leads both to feelings of alienation and inadequacy in the son’s personal and masculine identity, and to internalized homophobia (Drescher, 1998).

During clinical practice, the psychoanalyst may encounter an adult patient with depressive symptoms and personality problems. Here, as shown in the first vignette, an unconscious conflict exists between the alienated self and the defended authentic self—caused by incongruent mirroring. This unconscious conflict leads to feelings of internalized homophobia, as well as feelings of inadequacy in masculine and personal identity.

#### Vignette 1

Robert is a 28-year-old homosexual male who came into psychoanalytic treatment for depressive symptoms and narcissistic personality features. He was the youngest of three children. As a child of kindergarten-age, Robert already regarded himself as being different from other boys. He experienced silent same-gender wishes, and felt unable to live up to his father’s expectations. His father strove to strengthen his son’s masculinity and popularity with other boys. Robert enjoyed sports, especially basketball. He became a player with the local, premier team. Yet his father remained dissatisfied with his son’s achievements in sports, and pushed him toward a better way – the father’s own more competitive, macho way—of playing.

During puberty, Robert struggled to come out openly. His father felt personally hurt and attacked in his paternal role, and responded with a rejecting attitude. Eventually Robert did well academically, and grew up to become a psychologist. Still, Robert developed personality problems, along with feelings of depersonalization, and depressive symptoms. Through long-term treatment, Robert gradually became conscious of the origins of his internal homophobia, as well as of his alienated, weak, personal and masculine identity. He became able to perceive how he had undergone incongruent mirroring by his father and peers. The result? Robert was able to distance himself. Thanks to affirmative treatment by his psychoanalyst, Robert developed a more authentic, confident self.

In this vignette the authentic homosexual, masculine and personal self was defended in childhood by devaluation and rejection as central interpersonal affective pattern (Lemma et al., 2011). In the psychoanalytic relationship, first the defended authentic self was made conscious and then new congruent secondary self-representations were developed by acknowledgement and affirmation in changing the core interpersonal affective scheme.

During puberty, the level of sex-steroid hormones rises strongly, leading to the emergence of adult sexual desires. The awareness of erotic sexuality and of the object of sexual desire starts in late latency. Such awareness develops within an oedipal-representational framework. By early adolescence, the development of the ownership of the sexual body—with adult masturbation and sexual fantasies—is intense, but mostly secretive and concealed. Sexual-fantasy programming consolidates with the formation of adult-erotic fantasies or love-maps. The erotic fantasies of adult heterosexual and homosexual males end up being both similar and varied (Friedman and Downey, 2008).

During adolescence, the emergence of conscious, homosexual fantasy fosters the development of adult homosexual identity. About 20% of homosexual youth report personal distress while coming out. Peer harassment is experienced by 50% of homosexual youth, especially in early and mid-adolescence. Homosexual men are confronted by the risk of coming-out distress and by the challenge of creating their own lives within the context of a non-normative, developmental pathway. Yet such challenges may also lead to new opportunities.

### DEVELOPMENT OF GENDER IDENTITY

The development of gender identity starts at birth with gender assignment. By age one-and-a-half, core-gender identity is formed: the consciousness of being a boy or girl. Curiosity about gender difference is visible during childhood, sexual-explorative play. For a boy, the psychosocial construction of a solid, masculine identity occurs in childhood during the oedipal situation by affirmative interaction with his parents, and again in adolescence specifically during affirmative interaction with his peers (Stortelder and Ploegmakers-Burg, 2010).

Gender-role behavior in boys frequently involves rough and tumble play (RTP). RTP involves acts of dominance, aggression, and competition. Gender-role behavior in girls typically involves maternal-doll play and princess-doll adornment with jewelry, make-up, and feminine clothes. There is asymmetry between the sexes in the tolerance of gender non-conformity by peers. Tomboys are likely to be accepted by both boys and girls, while gender non-conformant boys, e.g., “sissies,” are depreciated and bullied (Friedman and Downey, 2008).

Throughout childhood, girls are more drawn to the company of single female best-friends. During play, girls tend to be more verbal, empathic, and relational. Female, juvenile-peer groups are smaller, more flexible, and more organized around mutual empathic communication in durable relationships (Ibid).

During early adolescence, gender segregation in peer groups is an almost universal phenomenon. Male peer groups tend to be hierarchically organized and larger than female groups, consisting of up to 5–8 individuals. In play, juvenile boys tend to be dominant and competitive, devaluing behavior deemed weak or non-masculine. The gender-role insecurity boys experience motivates them to become defensively homophobic. In juvenile-male groups, terms such as “homosexual,” “queer,” and “faggot” connote behavior considered weak or feminine—rather than behavior related to sexual identity *per se* (Ibid). Boys accepted by their peers experience increased masculine security. Boys rejected by their peers experience extremely low status within their group, and undergo a sense of shameful, masculine inadequacy.

During clinical practice, the psychoanalyst may encounter an adult patient with homosexual wishes and a severely vulnerable, masculine identity. Here, an unconscious conflict exists between the alienated self—comprised of homosexual wishes serving as a defense against a vulnerable, masculine and procreative identity—within a heterosexual patient. This second vignette shows how such unconscious conflict leads to depressive symptoms and personality problems.

#### Vignette 2

Andy, a 32-year-old lawyer, has been in a relationship with a 30-year-old female nurse for a year. Andy is referred for psychoanalytic treatment due to personality problems and depressive symptoms. He reports having problems in sexual identity development, along with preoccupation and doubts about being homosexual. During his assessment, Andy discusses his strong preoccupation with developing a homosexual identity. He expresses doubts about continuing the relationship with his girlfriend, which he discusses openly with her. Andy goes out looking for romantic relationships with homosexual friends. Though he had relationships with a few women before his girlfriend, he had often felt inadequate. His previous girlfriend, before the nurse, had dumped him in a painful way. Still, Andy’s erotic fantasies are not same-gender oriented.

During psychoanalytic treatment, Andy seems to have developed a strong sense of masculine insecurity and inadequacy, as well as doubts about the development of his procreative identity. He worries whether he can meet the responsibilities of marrying his girlfriend: founding a family, having children, becoming a father. Andy reports that he had been regularly beaten up by his stepfather since early childhood. This man came into his life when he was 1 year old, his biological father being absent. Andy, the youngest of three brothers, was a small and weak child. He was bullied by peers. He had also been humiliated by his two bigger and stronger brothers. Upon reaching puberty, his brothers had forced him to grant them sexual favors. Recalling his youth, Andy says his only special, affirmative remembrance is of a high-school teacher who positively mentored him and served as a father substitute.

During psychoanalytic treatment, Andy demonstrates a strong “father hunger.” Through the psychoanalytic relationship, Andy becomes conscious of how his alienated and weak masculinity is connected to those devaluing experiences with his stepfather, peers, and older brothers. Andy gradually distances himself from feelings of devaluation. He looks to unconsciously repair his vulnerable, masculine self through the affirmation and admiration of homosexual friends. Thanks to the father-transference with his psychoanalyst, who serves as a new, developmental object, Andy is able to create a solid, masculine, heterosexual identity. Eventually Andy marries his girlfriend and fathers two children.

In this vignette the authentic heterosexual, masculine, personal and procreative identity was defended in childhood abuse and humiliation as central interpersonal affective pattern (Lemma et al., 2011). In the psychoanalytic relationship, first the defended authentic self was made conscious and then new congruent secondary self-representations were developed by fathering and strengthening in changing the core interpersonal affective scheme.

Studies report that many homosexual men and women experienced gender non-conformity during childhood. Cohler and Galatzer-Levy (2000) emphasize how sexual identity (erotic fantasy) and gender-role behavior (masculinity and femininity) are not necessarily related. Yet the anatomical sites that regulate sexual behavior are distinct from the anatomic sites that influence gender-role behavior (Friedman and Downey, 2008). Strong, cross-gender interest during childhood is linked with homosexuality to a certain degree, yet it cannot predict homosexual identity. Many gender-non-conforming children become heterosexual. According to a clinical study of 77 children with gender identity disorder (GID) during adolescence, 20% of the GID children persisted and applied for sex-reassignment, while 80% ceased being gender dysphoric. Within this desistence group, about half of the boys and all of the girls with childhood GID developed in an adult, heterosexual direction (Wallien and Cohen-Kettenis, 2008). Homosexual men likewise demonstrate great diversity in gender-role behavior from the hyper-masculine (“leather men”) to hyper-effeminate (“drag queens”), to displays of average masculine behavior (Cohler and Galatzer-Levy, 2000).

### DEVELOPMENT OF PROCREATIVE IDENTITY

Erikson (1968) describes the central psychological developmental task of middle adulthood as the progression to generativity versus stagnation in self-absorption. Generativity involves beliefs and actions which express concern for the next generation’s welfare. The procreative identity demonstrates how a human being perceives and expresses consciousness as an individual creating and caring for offspring. The common way of caring for the next generation is through biological procreation. Darwin (1859) introduced the evolutionary theory of biological procreation as the transmission of genes to the next generation. Darwin (1859) emphasized the importance of continuity of the species; how continuous genetic variation and natural selection are the sources and driving forces of evolution. In the struggle for life, genetic variation or biodiversity makes humans flexible enough to adapt to an ever-changing environment.

From an evolutionary perspective, homosexuality has been continually present in many cultures and animal species. Bagemill’s (1999) research shows that homosexual behavior occurs in 300 vertebrate species. According to numerous, cross-cultural studies, homosexual behavior appears to be present in many cultures, such as the tribe of the Sambia in New Guinea, and in ancient cultures of Greece, Japan, and China (Aldrich, 2006). But what is the evolutionary, adaptive benefit of biologically non-reproducing homosexuals?

One major theory suggests that cultural evolution occurs alongside biological evolution (Dawkins, 1976). Cultural evolution follows principles similar to biological evolution. Elements of culture—e.g., ideas, skills, faiths, and science—are copied and transmitted to the next generation, from brain to brain. According to Dawkins (1976), cultural evolution has gradually influenced biological evolution. The human brain has grown in size, from 500 to 1500 g, in the service of cultural evolution.

Procreative consciousness is present in children from age five. In a clinical child-psychiatric assessment, most children spoke positively about whether they as adults would like to get married and have children. Research shows that during adolescence this procreative consciousness is equally present in young homosexual and heterosexual men (Berkowitz, 2007). Some homosexual men and women shape their generativity by means of biological procreation, others by means of cultural procreation. The masculine, procreative identity involves becoming a father, and developing the capacity for paternal, watchful protectiveness (Diamond, 1997).

Today, a significant number of openly homosexual men have children by committing to a parental alliance with a lesbian couple, or by surrogacy or adoption. Many family modes of differing, parental alliances of lesbian mothers and homosexual fathers exist. Such alliances vary: from co-parenting, to visiting schemes of one-to-several days a week and alternate weekends. Such constellations of parenting leave open the possibility that a child may later wish to explore the genetic contribution of both parents to his constitutional self and to his identity development (Golombok and Tasker, 2010).

Back in the 1970s, researchers studying homosexual parenthood questioned whether children growing up in homosexual families were at special risk for developing psychological problems and gender disturbance. In these studies, no differences in psychological development between children of homosexual and heterosexual parents were found. More recent studies indicate that lesbian and gay families—being very much pre-planned—report better-quality partner relationships and better relationships with children than in the heterosexual population, where unintended pregnancies occur far more frequently (Goldberg, 2010). However, the psychological outcome for adult children of gay and lesbian parents from a prior generation—specifically an adult who is the product of a parent’s early, failed, heterosexual union—is poorer than the psychological outcome for adult children from intact heterosexual families. Why? They not only had to deal with the disclosure process of their homosexual parent, but with their parents’ marital conflict and divorce, and with their own reorientation following the family break-up, along with loyalty conflicts and fear of abandonment (Goldberg).

A new wave of research is examining the dynamics of same-gender parenting, and how different kinds of homosexual family experiences have implications for child development (Telingator and Patterson, 2008). Such studies indicate that homosexual fathers focus particular attention on normative, gender-role socialization in their heterosexual sons. Adolescent children of homosexual parents may experience some degree of embarrassment, however, in regard to public exposure of their parents’ sexual identity.

In clinical practice, the psychoanalyst may encounter an openly homosexual father with a heterosexual son. The son’s questioning of his father–son relationship emerges in early adolescence. For the early-adolescent-heterosexual boy, unconscious conflict may arise. Such conflict will likely consist of fear of homosexual identity linked to age-related insecurity in masculine development. A temporary distancing may then occur between the heterosexual son and his homosexual father, leading to questions about the procreative identity of the homosexual father, as shown in this third vignette.

#### Vignette 3

John is a 45-year-old business man, who comes for consultation about questions regarding the relationship with his son and doubts about his role as a gay father. John has been in a relationship with his male partner for 20 years, and has been married for 8 years. He and his partner have a parental alliance with two lesbian mothers. John is the biological father of a son, Michael who is 13 years old and a daughter, age 10. Michael and his sister are from the same biological mother and father. Since birth, the children have lived with the mothers and visited the fathers 1 day per week and every other weekend. The mothers live nearby. Michael has developed well psychologically.

In regard to Michael’s psychosexual development, John noticed that Michael appeared to be developing in a heterosexual direction since kindergarten age. Michael showed some romantic interest in a girl in his class at age five, and had reveries about later marrying his mother. He had a girlfriend when he was 10. The father–son relationship developed positively, until Michael reached early adolescence, when some distancing in the relationship occurred. This distancing induced John’s worries about his procreative identity as a gay father. In psychoanalytic treatment, it appeared that Michael was experiencing an emergent, age-related, homophobic attitude within his male peer group. Because of peer-pressure, Michael developed a normal, temporary, homophobic attitude and felt unease—in his sexual identity and masculinity formation—about the relationship with his homosexual father. In psychoanalytic treatment, John started to understand the homophobic reactions of his son, as well as his own feelings of hurt and rejection, and learned how to handle them. The father–son relationship became normalized again after Michael’s early adolescence.

In this vignette first the authentic emerging heterosexual and masculine identity in the son is temporary defended by a homophobic attitude, which decreases with age because of further maturation and strengthening of the heterosexual and masculine identity. The authentic homosexual and procreative identity of the father is challenged and defended because of distancing and rejection. In the psychoanalytic relationship first the authentic self is made conscious and then restored by clarification and validation.

## CONCLUSION

In clinical psychoanalytic practice, several varieties of sexual identity development can be encountered as examples of unconscious conflicts between the alienated self-representations and the defended, authentic self. As shown in the first vignette, incongruent mirroring leads to feelings of alienation and of inadequacy in personal and masculine identity, along with internalized homophobia, in a homosexual patient. In the second vignette, homosexual wishes and questions about homosexual identity arise, as a defense against a vulnerable masculine and procreative identity, in a heterosexual patient. In the third vignette, fear regarding homosexual identity, linked to age-related insecurity in masculinity development, arises in early adolescence in a heterosexual boy. In homosexual parenthood, a temporary distancing emerges in early adolescence between the heterosexual son and homosexual father, leading to questions about the procreative identity of the homosexual father.

In the contemporary, neuropsychoanalytic model, the development of sexual identity is caused by the interaction of constitutional factors and psycho-social influences in which the cultural framework is also important. The model of Affect Mirroring provides a suitable model for the development of the self, as specified in personal gender-role, sexual and procreative identity. Incongruent mirroring leads to unconscious conflict between the defended, authentic self and alienated self representations. As demonstrated in all three vignettes, such unconscious conflicts can be expressed clinically in feelings of depersonalization, personality problems, depressive symptoms, and internal homophobia. Varieties of sexual identity development are often linked to difficulties in personal gender-role or procreative-identity development, also described by the vignettes. This psychoanalytic model, presented for the treatment of the non-authentic or alienated self in the psychoanalytic relationship, may be helpful in clinical practice, as demonstrated in the vignettes.

## Conflict of Interest Statement

The author declares that the research was conducted in the absence of any commercial or financial relationships that could be construed as a potential conflict of interest.
